# The child-to-family education program regarding self-medication: A theory-based interventional study

**DOI:** 10.34763/jmotherandchild.20222601.d-22-00009

**Published:** 2022-07-20

**Authors:** Fakhreddin Chaboksavar, Gholamreza Sharifirad, Razieh Pirouzeh, Farzad Jalilian, Narges Rafiei, Morteza Mansourian

**Affiliations:** Department of Health Education and Health Promotion, Babol University of Medical Sciences, Babol, Iran; Department of Health Education and Health Promotion, Islamic Azad University, Qom Branch, Iran; Department of Public Health, Esfarayen Faculty of Medical Sciences, Esfarayen, Iran; Research Center for Environmental Determinants of Health, Kermanshah University of Medical Sciences, Kermanshah, Iran; Health Management and Social Development Research Center, Golestan University of Medical Sciences, Gorgan, Iran; Health Management and Economics Research Center, Health Management Research Institute, Iran University of Medical Sciences, Tehran, Iran

**Keywords:** Child-to-family, Health Belief Model, self-medication, educational intervention

## Abstract

**Background and objective:**

Self-medication is considered one of the largest social, economic, and health problems in developing communities, including Iran. The present study aimed to determine the effectiveness of a child to family communication approach to self-medication based on the Health Belief Model [HBM].

**Material and methods:**

This study is a quasi-experimental study that was conducted on 124 students in the fourth grade and their mothers in the intervention and control groups in Isfahan city, Iran. Using multi-stage simple random sampling, two primary schools were selected from each group, and then one class was selected from each primary school. Students in the intervention group attended the educational sessions. Then, the students were required to transfer their education to their families. The impact of education on mothers was evaluated two months after the last session. Data were collected before and after the intervention with a researcher-created questionnaire based on the HBM and were analysed using the SPSS 17 software package, with chi-square, Mann-Whitney U, and Wilcoxon tests.

**Results:**

Before training, there was not a significant difference in the mean scores of the HBM constructs in terms of the demographic characteristics between the two groups. After the educational intervention, the mean of knowledge and HBM constructs were increased significantly, and the mean of perceived barriers decreased significantly in the experimental group [*P* < 0.001].

**Conclusion:**

According to the present study, the child-to-family education based on the HBM was effective. It is suggested that educational interventions based on the HBM be designed and implemented to decrease self-medication in Iranian families.

## Introduction

Self-medication is considered one of the largest social, economic, and health problems in developing communities, including Iran [1]. It is an attempt to solve health problems without seeking assistance from an expert [2]. Irrational or arbitrary use of medication can lead to various side effects, including microbial resistance, lack of response to treatment, and poisoning. Moreover, self-medication could disrupt pharmacology market causing exorbitant costs that increased per capita medicine costs in communities [3, 4, 5, 6, 7]. Today, it is known that 65% of diseases in Iran result from an incorrect prescriptions and irrational consumption of medicine. Self-medication rate in Iran is reported to be almost three times higher than the global average; therefore, Iran is among the world's top 20 countries in terms of self-medication, and it is the second in Asia after China [8]. The prevalence of self-medication varies from 18% of students [9], 35.4% of pharmacy visitors [10], 54% and 71% of people referred to health centers [11, 12], 76.9% of medical students [13], and 83.3% of in-patients [14]. The overall prevalence of self-medication in Iran is 53% [8], which is higher than in other studies conducted in China (32.9%, 2011) [15], Brazil (29.9%, 2010) [16], Nigeria (19.2%, 2011) [17], Portugal (18.9%, 2014) [18], and India (11.9%, 2014) [4].

The "child-to-child education" communication strategy has been used effectively since 1978. The underlying concept of child-to-child education is based on the UN Convention on the Rights of the Child (CRC) and children's participation in health promotion [19]. The child-to-child education concept as a learning process transfers children's knowledge to other children (from child to child), families (children to families), and society (children to society) [20].

At present, this educational model has been used in different countries and nations and has become popular in health education and prevention issues [21]. Research shows that children learn better and more effectively when they are actively involved in learning, and that information is more easily transmitted between people of the same age [22, 23]. This is the potential of children to teach others about the meaning of child-to-child support. The child-to-child approach encourages children to play an effective role in the community and provides children with a set of knowledge and abilities that foster self-esteem and sense of usefulness in children. It makes children ready to introduce various aspects of health messages based on predetermined goals to other children, families or communities. Child-to-child education not only helps educate young children and peers who have no chance of attending school, but it can be very effective in educating parents (child-to-family) and the community (child-to-community) [24]. In this regard, studies conducted with respect to child-to-child education [25] and child-to-family education [26] show the effectiveness of this educational method.

Schools, the most powerful social and educational organisations, have a significant impact on children, families, and communities. Almost all children spend a lot of time at school during their lives. Therefore, the development of children requires cohesive efforts by family, school, and their interrelationships. As a result, health messages can be passed on from teachers to children and from children to peers, siblings, and parents [25].

In view of the increased prevalence of self-medication in societies and the direct role of individuals in choosing and taking medications, to enable people to have a long, healthy, and active life, it is necessary to determine factors affecting behavioural change in achieving health behavior (self-medication avoidance). To this end, researchers have used health education and promotion models to identify the factors influencing behaviour change. One of these comprehensive and effective models used in health- behaviour science is the Health Belief Model (HBM). This model considers behaviour as a function of the knowledge and attitudes of individuals. According to its components, the HBM stems from an idea that leads to the perception of health threats by people and directly affects their behaviour with respect to health [27]. This can increase the perceived susceptibility and perceived severity in terms of arbitrary medication consumption. In view of the perceived barriers, interests, and guides, the HBM leads people to adopt healthful behavior. Therefore, this study used the child-to-family education approach regarding self-medication based on the HBM as an appropriate communication method to transfer knowledge from educated children to their families, especially their mothers and assessed the impact of the intervention.

## Material and methods

### Study design and population

This quasi-experimental study was conducted in Isfahan province, Iran, in 2017. For this purpose, two regions with similar demographic, economic, and social characteristics were selected and divided into intervention and control groups based on random allocation.

For this purpose, first, two regions with similar demographic, economic, and social characteristics were selected and based on simple random allocation, were divided into two groups of intervention and control so that one region was identified by the letter A and the other by the letter B. Then the words intervention and control were written on two separate papers and placed in a lottery container, and it was determined that each of the words intervention or control that came out belonged to zone A, and thus zone A was included in the control group; after that two schools were randomly selected from each area. Then two elementary schools were randomly selected from each region, after the one class selected from each primary school; therefore, a total of four classes from four primary schools randomly selected for participating in intervention and control group. In coordination with the education authorities in four primary schools, 62 students of fourth-grade and their mothers were selected in control group and 62 students in the fourth grade and their mothers in the intervention group.

To obtain the sample size, we used the following formula:


$$\begin{array}{ll} n= & \frac{2{\left[Z_{1-\alpha /2}+Z_{1-\beta }\right]}^{2}[p[1-p]]}{{\left[p_{1}-p_{2}\right]}^{2}} \end{array}$$

The confidence level and test power were 0.95 and 0.90 respectively. The *p*-values were *p* = 0.5 and *p*_1_-*p*_2_ = 0.3.

Thus, the sample size was 60 students. After considering potential dropouts, 62 students were selected in each group.

The students selected were asked to participate in the study. Mothers of the students were required to complete a questionnaire before the intervention. The content of the educational texts and communication strategy was developed based on the child-to-family education approach. Two months after the last training session, mothers were asked to complete the post test.

### Measuring tools

A questionnaire was used to collect the required data in three parts. The first part included five items about demographic information. The second part had 10 items about awareness. The third part of the questionnaire included five questions on perceived susceptibility, four on perceived severity, seven on perceived barriers, four on perceived benefits, and two questions on internal and external cues to action for self-remedy. The scoring of the questionnaire was as follows: in awareness items, the correct responses received a score of one and the wrong responses got a score of zero; at the end, the participant’s score was divided to the total score of the given part of the questionnaire multiplied by 100 to form a score of one hundred units.

Perceived susceptibility, perceived severity, perceived benefits, and perceived barriers were also measured on a five-point Likert scale ranging from strongly disagree [1] to strongly agree [5]. Overall, the total scores for each section (susceptibility, severity, barriers, and benefits) were calculated based on 100 scores. The questions of internal and external cues to action for self-remedy were calculated using frequency rate. The validity and reliability of the questionnaire has been confirmed by Shamsi et al. (Cronbach’s alpha = 0.84) [28]. Also in this study, the content validity method was used to determine the validity of the questionnaire. For this purpose, the questionnaire was approved by health education specialists, pediatricians, and general practitioners. Reliability was tested using a test–retest design with a two-week interval between measurements, and the Cronbach's alpha coefficient was 0.83.

### Intervention

In this study, the child-to-child communication method was conducted in an indirect training to intervention group with the aim to transfer educational content from students to their families. The curriculum designed for students included lecture sessions with questions and answers and using communication methods including contraction with peer groups, educational notes, educational videos, poster and pamphlet design, and wallpaper designed by students over six sessions lasted 60 min (Table 1).

**Table 1:** Summary of the education session structure based on HBM.

Sessions	Steps of the HBM	Educational content title
First	Awareness	Establish interactions between students, information about self-medication use through lectures
Second	Perceived sensitivity stage	Expression of individual and social complications caused by self-medication use, change in body function with the help of the group painting method in the form of wallpaper
Third	Perceived severity stage	Showing educational videos about the common side effects of self-medication use
Fourth	Perceived benefit stage	The benefits of proper medication use and avoidance of arbitrary use by peer group education
Fifth	Perceived barriers stage	Group discussion on ways to reduce self-medication use
Sixth	Review	Provide a summary of the content of the previous sessions

Also, in one of these activities, students were asked to write an essay on arbitrary medicine consumption and then transfer these issues to their families. Upon completion of the education program and ensuring the satisfaction of students' learning, the final evaluation of mothers was conducted two months later. It should be noted that the control group did not receive any educational programs related to arbitrary consumption of medicine during diagnostic evaluation before the intervention and evaluation of the effect of educational intervention; both intervention and control groups were asked to complete a questionnaire.

## Data analysis

Data were gathered and analysed using SPSS 17 software (Statistical Package for the Social Sciences). To compare control and intervention groups, the chi-square test was used with respect to the qualitative variables; the Mann-Whitney U test was used to compare control and intervention groups; and the Wilcoxon test was used to compare before and after intervention. A level of 0.05 was set as statistically significant.

## Ethical considerations

This study is the result of the MSc. thesis in health education. Ethical approval for this study was obtained from the Ethics Committee of the Isfahan University of Medical Sciences (IR.MUI.REC.392470). At all stages of the study, from data collection to the end of the analysis and reporting the findings, informed consent, anonymity, and the right to withdraw from the study at any time were observed to meet ethical requirements for this study. Besides, participants entered completely voluntarily and consciously, and their coordinates and personal info remained completely confidential.

## Results

The mean age of mothers was 32.20 ± 3.54 and 35.24 ± 4.47 years in the control and intervention groups, respectively. There was no significant difference between the two groups in terms of occupation, education level and health insurance coverage. Most mothers were housewives in terms of their occupational status. In all, 93.8% and 91.9% of the mothers had health insurance coverage in the control and intervention groups, respectively. With respect to education, most mothers had finished secondary school. There was no significant difference between the two groups for these variables (Table 2).

**Table 2:** Distribution of frequency of subjects in terms of occupation, education, and health insurance coverage.

Demographic characteristics		Intervention group		Control group		Chi-square test
		Frequency	Percentage	Frequency	Percentage	*P*-value
**Occupation**	Housewife	57	91.9	55	85.9	0.284
	Employed	5	8.1	9	14.1	
**Insurance coverage**	Yes	57	91.9	60	93.8	0.693
	No	5	8.1	4	6.3	
**Education**	Illiterate	0	0	1	1.6	0.218
	Primary	6	9.7	5	7.8	
	Middle	4	6.5	6	9.4	
	Secondary school	47	75.8	39	60.9	
	College	5	8.1	13	20.3	

Investigating the effectiveness of the child-to-family educational curriculum showed a significant difference between the levels of awareness, perceived susceptibility, perceived severity, perceived benefits, and perceived barriers of the mothers in the intervention group before and after the intervention; however, there was no statistically significant difference in the control group (*p*-value < 0.001). (Table 3)

**Table 3:** Comparison of the mean scores of awareness and HBM structures with respect to self-medication before and two months after educational intervention in the intervention and control groups.

Variables	Group	Before intervention	After intervention	*P*-value**
		Mean ± SD	Mean ± SD	
**Awareness**	Intervention	65 ± 14.1	82.5 ± 11.8	*P* ˂ 0.001
	Control	68.5 ± 15.1	65.1 ± 15.2	*P* = 0.056
	*P*-value*	*P* = 0.221	*P* ˂ 0.001	-
**Perceived susceptibility**	Intervention	63.4 ± 10.76	69.92 ± 9.76	*P* ˂ 0.001
	Control	63.04 ± 11.96	63.4 ± 11.76	*P* = 0.858
	*P*-value*	*P* = 0.572	*P* ˂ 0.001	-
**Perceived severity**	Intervention	70.7 ± 13.55	81.35 ± 13.5	*P* ˂ 0.001
	Control	74.6 ± 16.45	74 ± 17.3	*P* = 0.830
	*P*-value*	*P* = 0.059	*P* = 0.020	-
**Perceived barriers**	Intervention	48.45 ± 11.68	37.62 ± 11.4	*P* ˂ 0.001
	Control	47.62 ± 12.68	49.08 ± 14.51	*P* = 0.149
	*P*-value*	*P* = 0.790	*P* ˂ 0.001	-
**Perceived benefits**	Intervention	82.65 ± 11.15	93.85 ± 6.9	*P* ˂ 0.001
	Control	85.2 ± 12.85	84.6 ± 11.55	*P* = 0.298
	*P*-value*	*P* = 0.108	*P* ˂ 0.001	-

*Mann-Whitney U test. ** Wilcoxon Test.

The results also suggest that the physician had the most frequent external cues as to action with respect to self-medication for both intervention and control groups before and after the intervention; next was TV with a statistically significant decrease in frequency compared with the intervention in the intervention group (Table 4).

**Table 4:** Frequency distribution of external cues to action with respect to self-medication based on participants’ views before and two months after intervention in both groups.

	Intervention				Control			
	Before intervention		After intervention		Before intervention		After intervention	
Cues to action								
	Frequency	Percentage	Frequency	Percentage	Frequency	Percentage	Frequency	Percentage
**Physician**	56	90.3	61	98.4	58	90.6	60	93.8
**Family and peers**	3	4.8	0	0	5	7.8	7	10.9
**Books and pamphlet**	11	17.7	1	1.6	7	10.9	8	12.5
**Journals**	2	3.2	0	0	7	10.9	8	12.5
**Radio**	2	3.2	0	0	4	6.3	5	7.8
**TV**	18	29.0	3	4.8	15	23.4	16	25.0
**others**	4	6.5	0	0	1	1.6	3	4.7

The fear of side effects due to self-medication was the most frequent internal cue to action with respect to self-medication in both intervention and control groups before and after the intervention. Both lack of belief in self-medication and the family and peer's influences were shown as the next most frequent factors (Table 5).

**Table 5:** Frequency of distribution of internal cues to action with respect to self-medication based on participants’ views before and two months after the intervention in both groups.

Cues to action	Intervention				Control			
	Before intervention		After intervention		Before intervention		After intervention	
	Freque ncy	Percenta ge	Frequen cy	Percenta ge	Frequen cy	Percenta ge	Frequen cy	Percenta ge
**Fear of side effects due to Self-medication**	44	71.0	37	59.7	47	73.4	43	67.2
**Lack of belief in self-medication**	12	19.4	22	35.5	8	12.5	14	21.9
**Family and peer influences**	6	9.7	3	4.8	9	14.1	7	10.9

## Discussion

This study aimed to determine the effectiveness of the child-to-family communication approach based on the HBM in Isfahan, Iran, emphasising self-medication, with the aim of promot behaviour that would prevent the problem. The study showed a significant difference in the awareness, perceived susceptibility, perceived severity, perceived benefits, and perceived barriers of mothers in the intervention group towards their arbitrary medicine consumption before and after the educational intervention; however, there were no significant differences in the control group which suggested the effectiveness of child-to-family education based on the HBM with respect to self-medication.

In the present study, the moderate mean score of awareness resulting from control and intervention groups before the intervention could be attributed to proper information of physicians and television. The mean awareness score in the intervention group significantly improved after the intervention, which contributed to results from Shamsi et al. [28, 29]. According to the results, the level of awareness of mothers of educated children is higher than mothers of uneducated children. Although many factors affect the level of health awareness, education seems to be the most influential one. It should also be noted that promoting healthful behaviours requires backgrounds that go beyond educating children and families.

The present study reports a statistically significant difference in perceived susceptibility after educational intervention between intervention and control groups which could be a meaningful indication of the effect of educational intervention on improving the perceived susceptibility of individuals in the intervention group. Most mothers after the educational intervention believed that they might also be exposed to self-medication.

With respect to perceived severity, the mean scores of both groups before the intervention were higher than the average level, which significantly increased after the intervention in the intervention group. Other studies have shown that warnings of the serious side effects of self-medication use and directing people’s attention to resulting health problems, as well as the high cost of treatment, have been important factors in improving individuals’ perceived severity [28].

Additionally, with respect to the benefits of avoiding self-medication, findings from the present study show that individuals’ perception about the benefits of proper medicine consumption was moderate in both intervention and control groups before the intervention, and it significantly increased in the intervention group after the intervention. It seems that mothers' attention to the fact that the correct use of medication reduces complications and thus that faster recovery from disease has an effect on improving the level of their perceived benefits.

The present study shows a statistically significant difference between the two groups in perceived barriers after educational intervention, indicating the impact of educational intervention on removing the perceived barriers to recommended medicine consumption in the intervention group, which was in line with other studies [28]. Also, interventions based on theories of health education and health promotion, which include various environmental, cultural, and behavioural dimensions, should be made to identify more barriers.

The results of this study indicate that students should never be considered as pure learners because they are able to transfer knowledge and health concepts to other individuals and groups. Moreover, the present study has confirmed that educating students about avoiding self-medication could improve awareness, perceived susceptibility, perceived severity, perceived benefits, and perceived barriers among their mothers. Studies in Iran have reported the essential role of the students in increasing the awareness of their parents [20, 26]. In other studies, conducted in Lao PDR, Madagascar, and Bolivia, the effectiveness of teaching students about self-medication in promoting their parents' own health behaviour has been proven [30, 31].

In the present study, physicians were the most important source of information for both the control and intervention groups, which was in agreement with the studies conducted by Hajji Seyed Javadi in Iran [32]. Neafsey et al. have also reported that nearly half of the participants (46%) to their study were informed by physicians [33]. Furthermore, the most important causes of self-medication among the population of both control and intervention groups included fear of side effects of self-medication, which could explain perceived susceptibility in mothers that was consistent with results from Pirzadeh and Sharifirad [34].

## Conclusion

The child-to-family education approach as an influential potential in promoting health issues amongst families and communities might employ students to play an important role in using strategies to change behavioural health. The results of the study on educational intervention based on the HBM showed the positive effect of education on awareness, perceived susceptibility, perceived severity, perceived benefits and perceived barriers, and, more importantly, on reducing self-medication of the study participants. Therefore, it is recommended that other centres make use of this model to reduce self-medication.
